# Oral hymecromone decreases hyaluronan in human study participants

**DOI:** 10.1172/JCI157983

**Published:** 2022-05-02

**Authors:** Joelle I. Rosser, Nadine Nagy, Riya Goel, Gernot Kaber, Sally Demirdjian, Jamie Saxena, Jennifer B. Bollyky, Adam R. Frymoyer, Ana E. Pacheco-Navarro, Elizabeth B. Burgener, Jayakumar Rajadas, Zhe Wang, Olga Arbach, Colleen E. Dunn, Anissa Kalinowski, Carlos E. Milla, Paul L. Bollyky

**Affiliations:** 1 Division of Infectious Diseases and Geographic Medicine, Department of Medicine,; 2 Division of Infectious Diseases, Department of Pediatrics,; 3 Department of Pediatrics,; 4 Division of Pulmonary, Allergy & Critical Care Medicine, Department of Medicine,; 5 Center for Excellence in Pulmonary Biology, Department of Pediatrics, and; 6 Advanced Drug Delivery and Regenerative Biomaterials Laboratory, Cardiovascular Institute & Pulmonary and Critical Care, Department of Medicine, Stanford University, Stanford, California, USA.; 7 Bioengineering and Therapeutic Sciences, UCSF School of Pharmacy, San Francisco, California, USA.; 8 Department of Anesthesiology and Operative Intensive Care Medicine (CCM, CVK), Charité-Universitätsmedizin Berlin, corporate member of Freie Universität Berlin, Humboldt-Universität Zu Berlin, Berlin Institute of Health, Berlin, Germany.; 9 Department of Epidemiology, Stanford University, Stanford, California, USA.

**Keywords:** Inflammation, Extracellular matrix

## Abstract

**BACKGROUND:**

Hyaluronan (HA), an extracellular matrix glycosaminoglycan, has been implicated in the pathophysiology of COVID-19 infection, pulmonary hypertension, pulmonary fibrosis, and other diseases, but is not targeted by any approved drugs. We asked whether hymecromone (4-methylumbelliferone [4-MU]), an oral drug approved in Europe for biliary spasm treatment that also inhibits HA in vitro and in animal models, could be repurposed as an inhibitor of HA synthesis in humans.

**METHODS:**

We conducted an open-label, single-center, dose-response study of hymecromone in healthy adults. Subjects received hymecromone at 1200 (*n* = 8), 2400 (*n* = 9), or 3600 (*n* = 9) mg/d divided into 3 doses daily, administered orally for 4 days. We assessed safety and tolerability of hymecromone and analyzed HA, 4-MU, and 4-methylumbelliferyl glucuronide (4-MUG; the main metabolite of 4-MU) concentrations in sputum and serum.

**RESULTS:**

Hymecromone was well tolerated up to doses of 3600 mg/d. Both sputum and serum drug concentrations increased in a dose-dependent manner, indicating that higher doses lead to greater exposures. Across all dose arms combined, we observed a significant decrease in sputum HA from baseline after 4 days of treatment. We also observed a decrease in serum HA. Additionally, higher baseline sputum HA levels were associated with a greater decrease in sputum HA.

**CONCLUSION:**

After 4 days of exposure to oral hymecromone, healthy human subjects experienced a significant reduction in sputum HA levels, indicating this oral therapy may have potential in pulmonary diseases where HA is implicated in pathogenesis.

**TRIAL REGISTRATION:**

ClinicalTrials.gov NCT02780752.

**FUNDING:**

Stanford Medicine Catalyst, Stanford SPARK, Stanford Innovative Medicines Accelerator program, NIH training grants 5T32AI052073-14 and T32HL129970.

## Introduction

Hyaluronan (HA), an extracellular matrix glycosaminoglycan, plays an important role in inflammation (1, 2). Within injured and infected tissues, HA is produced by stromal cells in response to inflammatory signals (3). At these sites, HA promotes immune activation (4–7), cellular migration (8, 9), and glycolytic metabolism (10). HA is implicated in immune dysregulation (11), cancer (12), and fibrosis (13) in diverse tissues and disease contexts.

In the lung specifically, HA accumulation is associated with a number of inflammatory diseases (14–19). HA is elevated in the bronchoalveolar lavage (BAL) fluid of patients suffering from chronic obstructive pulmonary disease (COPD) (20), interstitial pulmonary fibrosis (IPF) (21), and acute respiratory distress syndrome (ARDS) (22–27). In these settings, HA stimulates a proinflammatory cascade (28) and contributes to respiratory pathophysiology, including fluid accumulation, airway plugging, and impaired oxygen exchange (29–31). Notably, there is an inverse correlation between the concentration of HA (BAL fluid and serum) and the pulmonary oxygenation index in patients with ARDS (25, 32). Together, these reports support a pathological role for HA in chronic lung diseases.

Recently, HA has been implicated in the pathogenesis of COVID-19. We and others have shown that HA is abundant in the lung tissue and sputum of deceased patients with COVID-19 compared with healthy patients (33–35). The genes encoding the 3 HA synthases (*HAS1*, *HAS2*, *HAS3*) are likewise significantly upregulated in the BAL fluid of COVID-19 patients (36). In addition, serum HA has been identified as an independent predictor of COVID-19 severity, including the risk of hospitalization, intubation, and mortality (37). Taken together, these data support the pathological role and potential therapeutic targeting of HA in respiratory diseases.

Given the role of HA in mediating COVID-19 and other diseases, there is great interest in developing therapeutic approaches to targeting HA. Currently no FDA-approved drugs specifically target HA. Hymecromone, also known as 4-methylumbelliferone (4-MU), an agent developed in the 1960s for biliary colic, inhibits HA synthesis in preclinical models (38). Directly, 4-MU is a competitive substrate for UDP–glucuronyl transferases (UGTs), depleting one of the HA precursors, UDP–glucuronic acid (39). Indirectly, 4-MU reduces the expression of mRNA transcripts involved in HA synthesis (40, 41). The main metabolite of 4-MU, 4-methylumbelliferyl glucuronide (4-MUG), has also been shown to be bioactive (42). 4-MU has demonstrated therapeutic potential in multiple animal models (4, 38, 43–48). In the animal lung, 4-MU reduces HA and ameliorates disease in mouse models of lung infection (49–51), lung metastases (52), pulmonary hypertension (53), and pulmonary fibrosis (23).

However, it has been unclear whether hymecromone inhibits HA synthesis in humans and at which doses. The systemic oral bioavailability of hymecromone is reported to be less than 3%, mostly due to extensive first-pass metabolism (40, 54). Furthermore, hymecromone is rapidly metabolized, mainly to 4-MUG (54, 55), and has a half-life of 28 minutes in humans (55, 56). It therefore was unclear that sufficient concentrations of drug were present to inhibit HA synthesis in target tissues. However, we recently reported that 4-MUG is bioactive (42), raising the possibility that oral 4-MU in fact reaches therapeutic concentrations when both 4-MU and 4-MUG are considered.

Here, we investigated whether oral hymecromone at daily doses ranging from 1200 mg/d to 3600 mg/d in divided doses administered 3 times a day for 4 days reduces HA concentration in the sputum and serum of healthy human volunteers. During the study treatment, we closely assessed the drug levels in the sputum and serum in these individuals after 4 days of treatment as well as their safety and tolerability of hymecromone.

## Results

### Enrollment/demographic characteristics.

**Figure 1:**
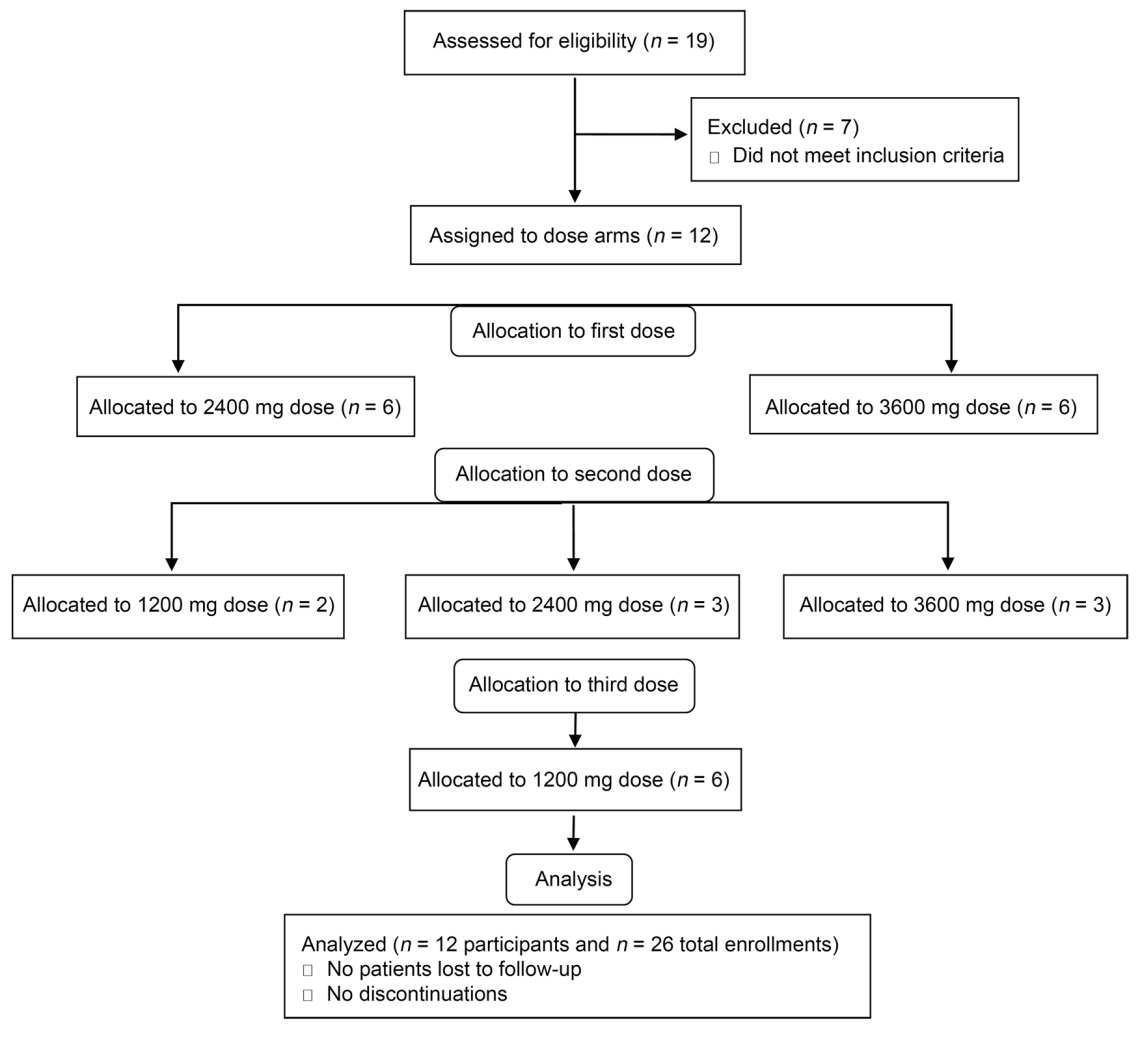
CONSORT diagram of study enrollment. Open-label, single-center, nonrandomized, dose-response study of hymecromone in healthy adults. Participants were assigned to receive hymecromone at 1200 mg/d, 2400 mg/d, or 3600 mg/d. First, participants were assigned to 1 of 2 study arms: 2400 mg/d or 3600 mg/d; participants were assigned to dose arms in a sequential manner until 6 participants were enrolled in each arm. Second, participants were invited to reenroll to complete either 1 or 2 additional dose arms. The first 6 individuals who volunteered to reenroll for 2 additional doses were first assigned to the high-dose arm opposite of what they received in the first enrollment and then received 1200 mg/d for their third enrollment. Additional individuals who volunteered to reenroll for 1 additional dose were assigned to the 1200 mg/d arm.

In total, 19 individuals were screened for the study; 7 were excluded because they did not meet screening criteria. Twelve unique, healthy volunteers were enrolled in the study; 4 completed 1 dose arm, 2 completed 2 dose arms, and 6 completed all 3 dose arms, for a total of 26 enrollments (Figure 1). Eight, 9, and 9 individuals completed the 1200 mg, 2400 mg, and 3600 mg arms, respectively (Figure 1). All participants who consented to study procedures and initiated treatment completed the study. Participants ranged in age from 22 to 65 years old; 58.3% were male, and 41.7% were female. The demographics of the study population are shown in Table 1.

There were 26 enrollments overall, with 8, 9, and 9 individuals completing the 1200 mg, 2400 mg, and 3600 mg arms, respectively. The drug was given per os (PO) in divided doses administered 3 times a day for 4 days. Each individual could contribute to multiple arms, and therefore the demographic characteristics are described for each dose arm and for the 12 unique subjects overall (Table 1). There were no significant differences in demographic characteristics between dose arms.

### Safety and tolerability of hymecromone.

Across all 26 enrollments, 9 adverse events (AEs) were recorded. All AEs were mild to moderate and resolved without intervention. Eight were assessed by study investigators as possibly related to study medication and 1 as probably related. In the 3600 mg arm, 6 AEs were reported, including headache affecting 2 subjects and diarrhea, dizziness, insomnia, and nausea, each affecting a single subject. In the 2400 mg arm, 1 subject experienced a headache. In the 1200 mg arm, 1 subject experienced insomnia, and 1 subject experienced asymptomatic elevation in aspartate aminotransferase (AST) and alanine aminotransferase (ALT), with other liver function tests normal (Supplemental Table 1; supplemental material available online with this article; https://doi.org/10.1172/JCI157983DS1). There were no clinically significant changes in the complete blood counts or renal function tests (Supplemental Table 2). Overall, these results indicate that hymecromone was generally safe and well tolerated at these doses.

### Effects of hymecromone on sputum HA.

**Figure 2:**
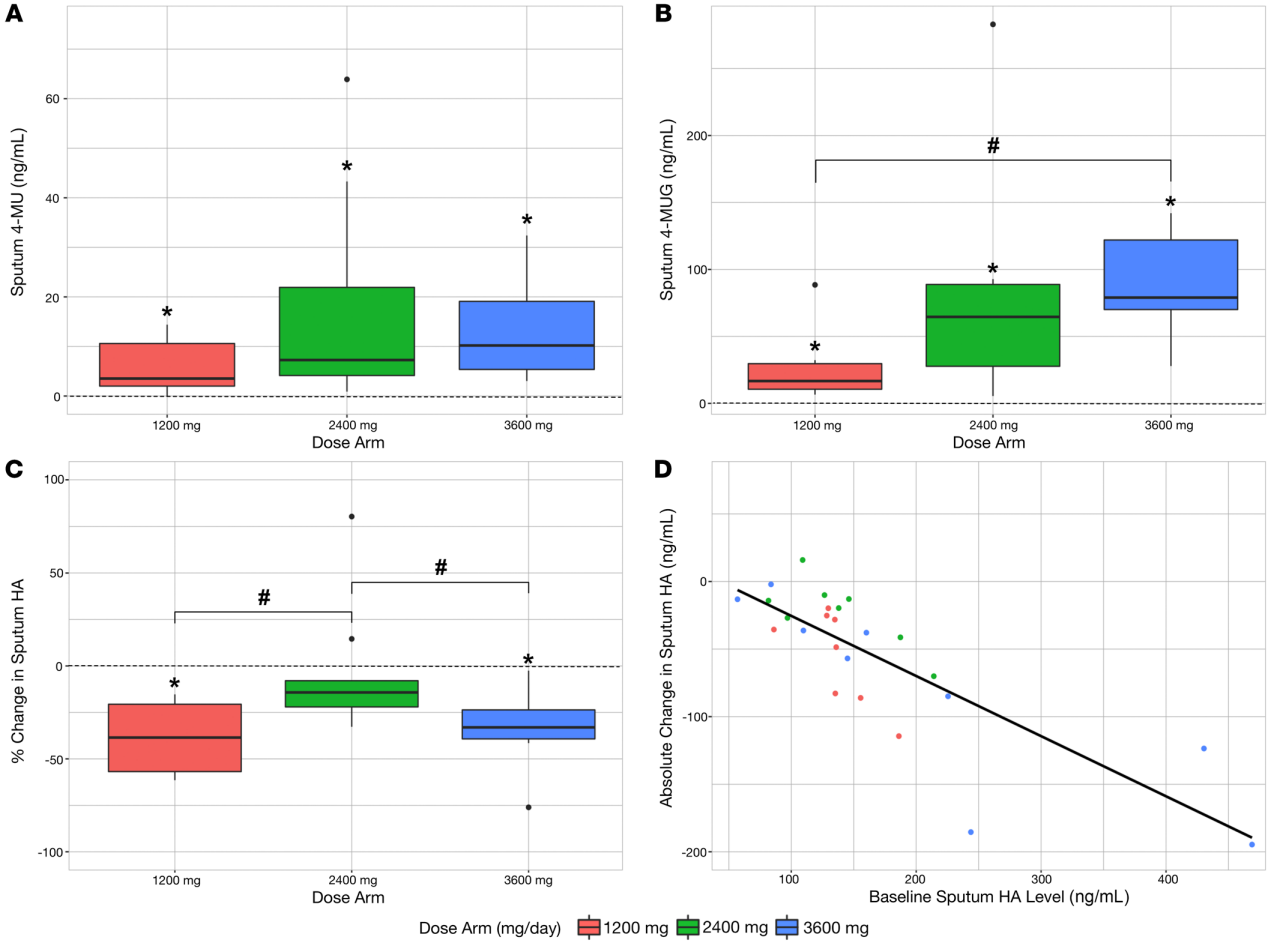
Sputum HA decreases after treatment with 4-MU. (**A**) Sputum 4-MU levels demonstrated a stepwise increase with increasing dose. One extreme outlier in the 2400 mg arm was removed from this graph. (**B**) Sputum 4-MUG levels demonstrated a dose-dependent increase. (**C**) Mean sputum HA decreased across all 3 arms. The change was statistically significant in the 1200 mg and 3600 mg arms. (**D**) Higher baseline sputum HA levels showed a greater response to treatment. *P < 0.05, difference from baseline to day 4 of treatment by paired *t* test; ^#^*P* < 0.05, difference between dose arms by unpaired *t* test. The dashed line indicates the baseline reference level. In all panels, *n* = 8, *n* = 9, and *n* = 9 for the 1200 mg, 2400 mg, and 3600 mg arms, respectively. Each boxplot represents the median, interquartile range, 1.5 times the interquartile range, and data points outlying the whisker range.

Sputum 4-MU and 4-MUG levels were significantly higher than baseline after 4 days of treatment across all dose arms. Sputum levels of 4-MU and 4-MUG increased with higher dosages, although the differences between dose arms were only significant between the 4-MUG levels of the 1200 mg and 3600 mg dose arms (Figure 2, A and B, and Table 2). Additionally, most drugs present in sputum existed as 4-MUG across study subjects. This is consistent with previous pharmacokinetics (PK) studies of hymecromone (54).

Sputum HA levels decreased from baseline to day 4 over the study period (mean absolute difference in HA: –46 ng/ml; 95% CI: –73.1, –19.0; mean percentage change in HA: –25.1%; 95% CI: –36.9%, –13.3%) (Table 2). Stratified by dose arm, this decrease was significant in the 1200 mg and 3600 mg arms (Figure 2C, Supplemental Figure 1A, and Table 2). The 3600 mg arm and 1200 mg arm showed a significantly greater decrease in sputum HA compared with the 2400 mg arm (Figure 2C and Table 2).

In the subanalysis restricted to the first enrollment of 12 individuals who received either 2400 mg/d or 3600 mg/d, the decrease in sputum HA was significantly greater in the 3600 mg arm than in the 2400 mg arm (Supplemental Figure 1B). In the subanalysis restricted to the 6 participants who reenrolled and completed all 3 dose arms, there was no significant difference in sputum HA levels by dose arm (Supplemental Figure 1C). For all individuals who reenrolled, baseline drug levels were rechecked at each enrollment and noted to be back to a background (near zero) level in all participants. To further evaluate for potential residual effects of reenrollment, mean baseline HA levels were evaluated and there were no statistically significant differences in baseline sputum HA levels across dose arms or different enrollments, although the variability did decrease with subsequent enrollments (Supplemental Figure 2, A and C).

Higher baseline sputum HA levels were associated with a greater decrease in sputum HA (Pearson’s correlation coefficient for absolute decrease: –0.59; 95% CI: –0.80 to –0.26) (Figure 2D). There was no association between changes in sputum HA and demographic characteristics, including sex, age, and BMI.

### Effects of hymecromone on serum HA.

**Figure 3:**
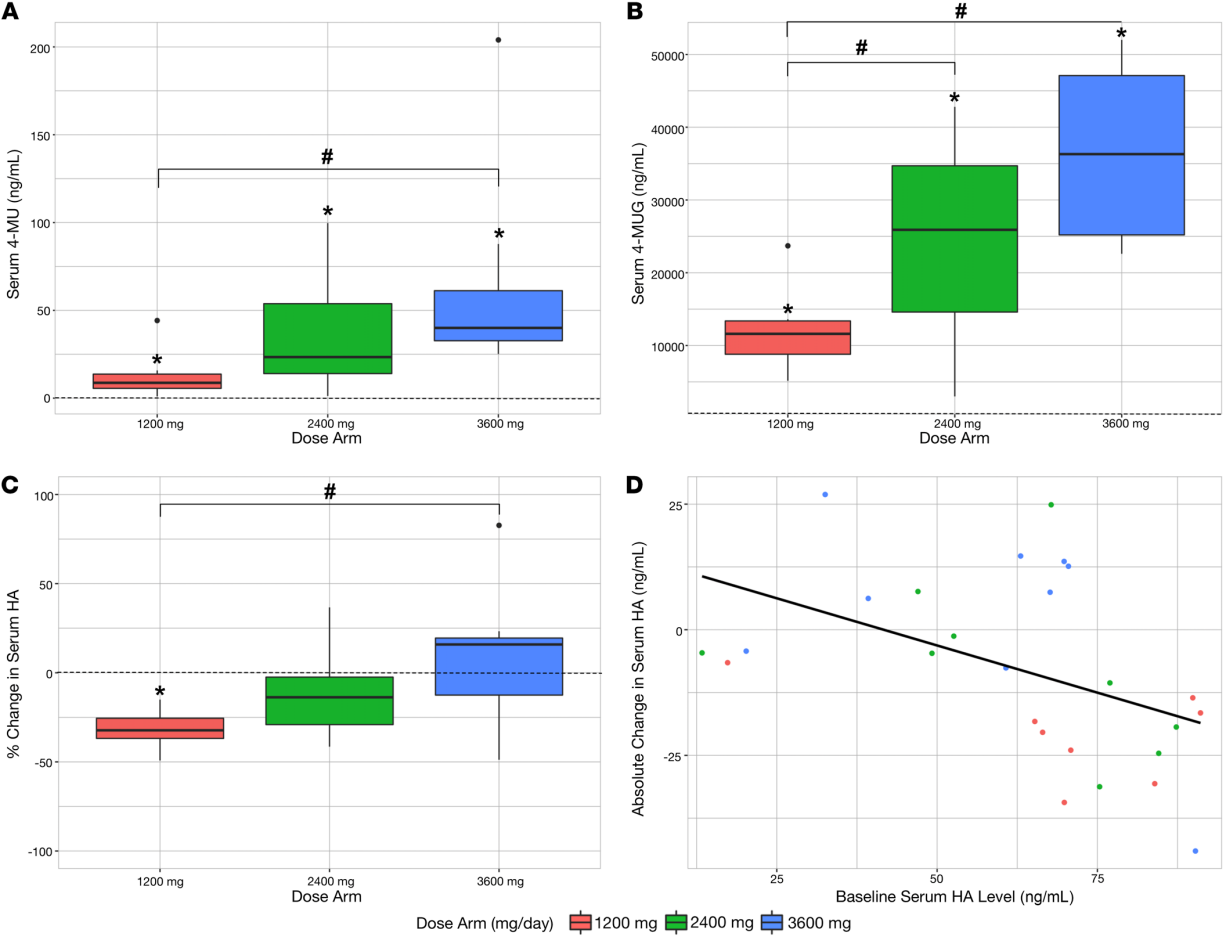
Serum HA decreases after treatment with 4-MU. (**A**) Serum 4-MU and (**B**) serum 4-MUG levels demonstrated a stepwise increase with increasing dose. (**C**) Serum HA decreased significantly in the 1200 mg arm only; this was also significantly different from the 3600 mg arm. (**D**) Higher baseline serum HA levels showed a greater response to treatment. *P < 0.05, difference from baseline to day 4 of treatment by paired *t* test; ^#^*P* < 0.05, difference between dose arms by unpaired *t* test. The dashed line indicates the baseline reference level. In all panels, *n* = 8, *n* = 9, and *n* = 9 for the 1200 mg, 2400 mg, and 3600 mg arms, respectively. Each boxplot represents the median, interquartile range, 1.5 times the interquartile range, and data points outlying the whisker range.

Serum levels of 4-MU and 4-MUG were significantly higher than baseline after 4 days of treatment across all dose arms. Serum 4-MU and 4-MUG levels increased with increasing hymecromone doses. This difference was significant for 4-MU for the 1200 mg versus 3600 mg 4-MU levels and for 4-MUG levels in the 1200 mg versus 3600 mg doses and the 1200 mg versus 2400 mg doses (Figure 3, A and B, and Table 2). Additionally, most drug present in serum existed as 4-MUG across study subjects, consistent with previous PK studies of hymecromone (54).

Overall, absolute serum HA concentration decreased from baseline to day 4 (mean absolute difference, HA: –7.8; 95% CI: –15.3, –0.3) (Table 2). Stratified by dose arm, this decrease was significant only in the 1200 mg arm (Figure 3C, Supplemental Figure 1D, and Table 2). The change in serum HA in the 1200 mg arm was also significantly different than in the 3600 mg arm (Table 2).

In the subanalysis restricted to the first enrollment, there was no significant difference in HA change between the 2400 mg and 3600 mg arms (Supplemental Figure 1E). In the subanalysis restricted to the 6 participants who completed all 3 dose arms, the percentage of HA change was significantly different in the 2400 mg arm (Supplemental Figure 1F).

Higher baseline serum HA levels were associated with a slightly greater decrease in serum HA (Pearson’s correlation coefficient for absolute decrease: –0.46; 95% CI: –0.72 to –0.09) (Figure 3D). There was no association between change in serum HA and demographic characteristics, including sex, age, and BMI. There were no statistically significant differences in baseline serum HA levels across dose arms or different enrollments (Supplemental Figure 2, B and D).

## Discussion

We report that hymecromone is safe and well tolerated in healthy human subjects at doses up to 3600 mg/d. Both sputum and serum drug concentrations increased in a dose-dependent manner, indicating that higher doses led to greater exposures. These data are consistent with nearly 50 years of clinical experience with this drug as a therapy for biliary spasm. We are aware of more than 60 completed clinical studies using hymecromone, including over 2600 participants with oral doses up to 2400 mg/d and treatment durations as long as 6 months, supporting the safety and tolerability of hymecromone (54, 57–64).

We further report substantial decreases in sputum HA concentrations after 4 days of hymecromone intake. We observed decreases in sputum HA concentrations across all dose arms, although this was only statistically significant in the 1200 mg and 3600 mg arms. The lack of statistical significance in the 2400 mg dose arm may be due to the large variance in HA levels in that arm. When evaluating only the first enrollment, we did see a dose response in the sputum. Arguably, this may be the most accurate, since it is not subject to possible confounding introduced by reenrollment. Yet we do not see this dose response when evaluating all the data or in the subanalysis of individuals enrolled in all 3 dose arms.

Serum HA concentrations also decreased substantially in the overall analysis and in the 1200 mg dose arm. The effect on serum HA was much less pronounced than in the sputum. This may be due to the fact that hymecromone targets HA synthesis, which occurs in tissues, such as the lungs, whereas basal serum levels reflect tissue catabolism, which is not affected by hymecromone. We saw a statistically significant difference between the 1200 mg and 2400 mg arms in the serum analysis evaluating the percentage change across all enrollments, but in none of the other analyses (absolute change or any of the restricted population analyses); this is likely because serum is reflective of a basal level HA level/lower threshold limit.

The 2 primary limitations of this study are the limited statistical power and the enrollment of healthy individuals. These limitations may explain why we did not observe a dose response in sputum and serum HA. The small sample size (<10 subjects per arm) limited our power to detect differences between dose arms, particularly at doses within a relatively narrow range. This power limitation was also evident in our analysis of the PK. Although we did observe a dose-dependent increase in 4-MU and metabolite 4-MUG levels, the difference was significant predominantly between the most disparate doses, 1200 mg versus 3600 mg, with limited power to detect smaller exposure-response differences. Another limitation is that we measured a single concentration at 90 minutes after doses. Single sampling is sensitive to variation in absorption and PK and therefore does not necessarily represent the peak concentration in each individual. Further studies are necessary to more clearly describe hymecromone PK.

The enrollment of healthy individuals with normal baseline HA levels likely also limited our ability to detect a dose-dependent response. HA concentrations may have a natural lower physiologic limit or basal rate, whereby increasing doses were unable to demonstrate a greater effect in this trial of healthy individuals. Supporting this assumption, we observed that higher baseline sputum HA concentrations were associated with greater decreases in sputum HA. Given that a more dramatic response was observed in participants with a higher baseline HA concentration, even at these relatively low HA concentrations, we hypothesize that hymecromone will have a meaningful effect in patients with pathologically elevated HA significantly higher than the healthy population. In disease states, HA concentrations are frequently elevated hundreds to several thousand times the normal range (22–27, 65). It is therefore plausible that individuals with disease might demonstrate a greater absolute response. It is also possible that these findings are a result of regression to the mean and further work is needed to determine the optimal dose in these disease states. Overall, our findings suggest that at the doses evaluated here, which have shown excellent safety and tolerability, we may expect to see good clinical efficacy.

Another potential limitation of our study is the crossover design in which individuals could reenroll in other dose arms. Although the potential unobserved impact of enrollment cannot be completely negated, we suspect that enrollment effect had minimal impact on our findings for several reasons. First, the patients who reenrolled in multiple dose arms had a washout period of at least 7 days, which is significantly longer than the half-life of 4-MU or 4-MUG (54). As an important control, we also observed that 4-MU and 4-MUG drug levels returned to baseline in all subjects prior to administration of the new dose. Additionally, baseline sputum and serum HA levels were remeasured prior to each new enrollment, and there was no significant difference in baseline sputum and serum HA levels stratified by dose arm or by enrollment. Furthermore, the reenrollment scheme allowed us to evaluate the relationship both between and within subjects.

This is the first study, to our knowledge, to assess the effect of hymecromone on HA in humans, highlighting the potential to repurpose this drug for inhibition of HA production in several acute and chronic pulmonary diseases. Further work is needed to evaluate the pharmacodynamics of 4-MU and 4-MUG in patients with elevated HA levels and to establish the optimal dose in pulmonary disease. Moreover, additional clinical trials are also needed to evaluate the clinical efficacy of 4-MU and 4-MUG in pulmonary diseases characterized by elevated HA. In conclusion, our study lays the groundwork for evaluating hymecromone in larger, randomized and controlled human clinical studies particularly targeting pulmonary diseases.

## Methods

### Study design.

We conducted an open-label, single-center, nonrandomized, dose-response study of hymecromone in healthy adults. Participants were assigned to receive the study medication for 4 consecutive days at 1 of 3 dose levels: 400 mg PO 3 times per day (1200 mg/d), 800 mg PO 3 times per day (2400 mg/d), or 1200 mg PO 3 times per day (3600 mg/d). In the first stage of the study, participants were assigned to 1 of 2 study arms: 2400 mg/d or 3600 mg/d; participants were assigned to dose arms in a sequential manner until 6 participants were enrolled in each arm. After a trial protocol modification, a second stage of the study commenced in which participants were invited to reenroll to compete either 1 or 2 additional dose arms. The first 6 individuals who volunteered to reenroll for 2 additional doses were first assigned to the high-dose arm opposite of what they received in the first enrollment (e.g., participants who received 2400 mg/d in the first enrollment would receive 3600 mg/d in the second enrollment) and then were assigned to received 400 mg PO 3 times per day (1200 mg/d) for their third enrollment. Additional individuals who volunteered to reenroll for 1 additional dose were assigned to the 1200 mg/d arm. This scheme was designed to optimize the comparison between the 2400 mg/d and 3600 mg/d arms, allow for intrasubject comparison, and result in roughly equal numbers of participants in all 3 dose arms. All reenrollments occurred after a washout period of at least 7 days from the end of the prior dose regimen.

### Participant population.

The study enrolled healthy adult volunteers. Individuals who were included in the study were between 18 and 65 years of age with no active medical problems or striking chronic diseases, had a normal BMI (18.5 — 30 kg/m^2^), and were not taking any other medications. Individuals were excluded if they had a history of any of the following: gastrointestinal disease including gastroesophageal reflux disease, gastritis, peptic ulcer disease or dyspepsia, dysphagia, achalasia, or difficulty swallowing capsules, tablets, or pills. Individuals were screened prior to enrollment and were excluded if they had elevated liver function tests, renal function tests, ECG abnormalities deemed clinically significant by the study physician, ongoing alcohol or drug use, were pregnant, lactating, allergic to any component of the study drug, or participating in another clinical trial. Participants were compensated 100 USD for their time for each study visit.

### Summary of treatment regimen and assessments.

Participants who met the screening criteria and consented to participate were enrolled in the study. On day 1, participants underwent a baseline sputum induction and blood draw. They were then administered their first dose of study medication and underwent a second blood draw 90 minutes later. Participants then took the study medication 3 times a day for 4 consecutive days. Participants were instructed to take the study drug with 250 ml water and with meals or a snack. On day 4, they returned to the clinic for another sputum induction and 2 blood draws taken before and 90 minutes after the 11th dose in clinic. HA concentrations were measured in the first and last blood samples and in the 2 sputum samples. 4-MU and 4-MUG concentrations were measured in all 4 blood samples and in both sputum samples. Participants recorded a daily diary of when they took their doses and any possible side effects noticed. A complete blood count, complete metabolic panel, and ECG were also evaluated at screening and on day 4. Final follow-up was completed by online survey 7 days after finishing the study medication.

### Analysis of HA concentration.

Sputum samples were treated with 250 U benzonase for 30 minutes at 37°C for nucleic acid digestion, followed by an incubation with 1 mg/ml proteinase K overnight at 65°C for further digestion. Proteinase K was heat inactivated by incubating the samples at 95°C for 30 minutes. Insoluble material was removed by centrifugation at 10,000*g* for 10 minutes before further processing. HA concentration was determined using a HA ELISA (Echelon Biosciences) following the manufacturer’s instructions.

### Analysis of 4-MU and 4-MUG concentration.

Liquid chromatography–tandem mass spectrometry (LC-MS/MS) was used to analyze 4-MU and 4-MUG concentrations in the serum and sputum samples of the study participants. 4-MU–13C4 (Toronto Research Chemicals) was used as the internal standard (IS) for 4-MU and 7-hydroxycoumarin β-d-glucuronide (Toronto Research Chemicals) as the IS for 4-MUG. The neat stock solutions of 4-MU and 4-MUG were mixed and diluted in 50% methanol to prepare spiking solutions ranging from 2 ng/ml to 5000 ng/ml for each compound.

For calibration standards, 25 μl of blank human serum or sputum was mixed with 25 μl of the spiking solutions. For samples to be tested, 25 μl of serum or sputum was mixed with 25 μl of 50 % methanol to make up the volume, and 25 μl of a mixture of the 2 IS (1000 ng/ml each in 50 % methanol) was then added. After vortexing all standards and samples, 150 μl of methanol/acetonitrile 20:80 (v/v) was added to the mixture and the sample was further vortexed vigorously for 1 minute followed by centrifugation at 1000*g* for 10 minutes; 100 μl of the supernatant was taken and diluted with 200 μl of Milli-Q water.

The LC-MS/MS system consists of an AB SCIEX QTRAP 4000 mass spectrometer linked to a Shimadzu UFLC system. Mobile phase A is HPLC grade water with 10 mM of ammonium acetate. Mobile phase B is HPLC grade acetonitrile. LC separation was carried out on a XSelect CSH C18 column (Waters Corp.) (3.5 μm, 4.6 × 100 mm) with gradient from 15% to 85% mobile phase B at 5 minutes, then from 85% to 95% mobile phase B at 8 minutes, and from 95% to 15% mobile phase B at 8.1 minutes. The analysis time was 10 minutes with a flow rate of 0.4 ml/min at room temperature; 20 μl of the extracted sample was injected. The mass spectrometer was operated in the negative mode with the following multiple-reaction monitoring (MRM) transitions: *m/z* 174.7→132.9 for 4-MU, *m/z* 178.7→134.9 for 4-MU-13C4 (IS), *m/z* 350.8→174.9 for 4-MUG, and *m/z* 336.9→160.9 for 7-hydroxy coumarin β-d-glucuronide (IS). Data acquisition and analysis were performed using Analyst, version 1.6.1, software (AB SCIEX).

### Statistics.

The study was analyzed by an intention-to-treat strategy. Safety labs, HA concentration, and 4-MU and 4-MUG concentrations were described using mean, SD, minimum, and maximum. Changes in labs from baseline to day 4 were compared using paired *t* tests. The differences in the changes in HA across different dose arms were compared using unpaired *t* tests for the primary analysis. A subanalysis comparing the HA change between the 2400 mg/d and 3600 mg/d arms restricted to the first enrollment was performed using unpaired *t* tests. A subanalysis comparing the HA changes across the 3 dose arms restricted to the 6 individuals who completed all 3 dose arms was performed using paired *t* tests. Sensitivity analyses were performed both including and excluding extreme outliers and Wilcoxon’s signed-rank tests. Statistical significance was based on 2-tailed tests with α of 0.05. Relationships between change in HA and baseline HA levels were also evaluated by Pearson’s correlation coefficients. Analyses were performed in SAS and R.

### Study approval.

This study was approved by the FDA and the Stanford University Institutional Review Board (IRB-43805) and was registered at ClinicalTrials.gov (NCT02780752). All healthy adult volunteers provided written, informed consent prior to participation in the study.

## Author contributions

JIR, NN, AK, and PLB designed the research study. JIR, NN, RG, GK, JS, ARF, AEPN, EBB, CED, CEM, and PLB conducted the research study. JIR, NN, RG, GK, SD, JS, JBB, ARF, AEPN, EBB, JR, ZW, OA, CED, AK, CEM, and PLB acquired and analyzed the data. JIR, NN, SD, and PLB wrote the first draft of the manuscript. All authors participated in interpreting the data and editing the manuscript. JIR, NN, and PLB wrote the final version of the manuscript. JIR and NN share the first author position, reflecting their equivalent contributions, with JIR listed first based on leading the clinical implementation.

## Supplementary Material

Supplemental data

ICMJE disclosure forms

## Figures and Tables

**Table 2:**
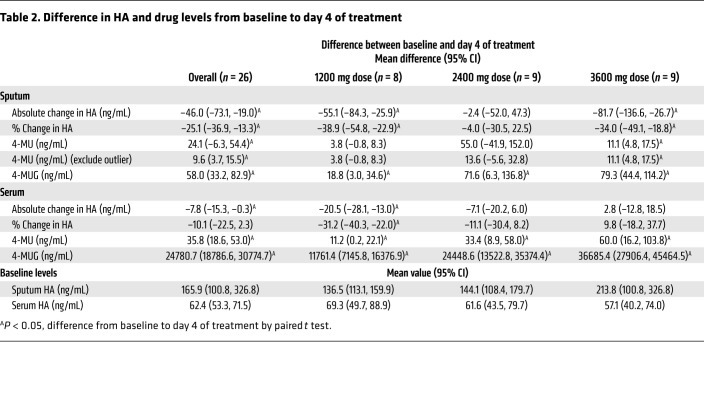
Difference in HA and drug levels from baseline to day 4 of treatment

**Table 1:**
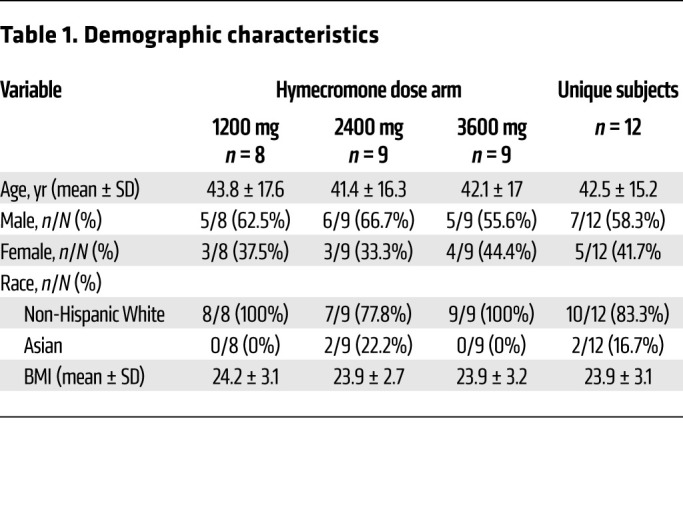
Demographic characteristics

## Version 1 05/02/2022

Electronic publication
