# Supplementary material for: Oral hymecromone decreases hyaluronan in human study participants
Source: J Clin Invest. 2022 May 2;132(9):e157983. doi: 10.1172/JCI157983 (PMC9057598; doi:10.1172/JCI157983)
Supplement: Supplemental data [file jci-132-157983-s197.pdf]

Supplemental Material

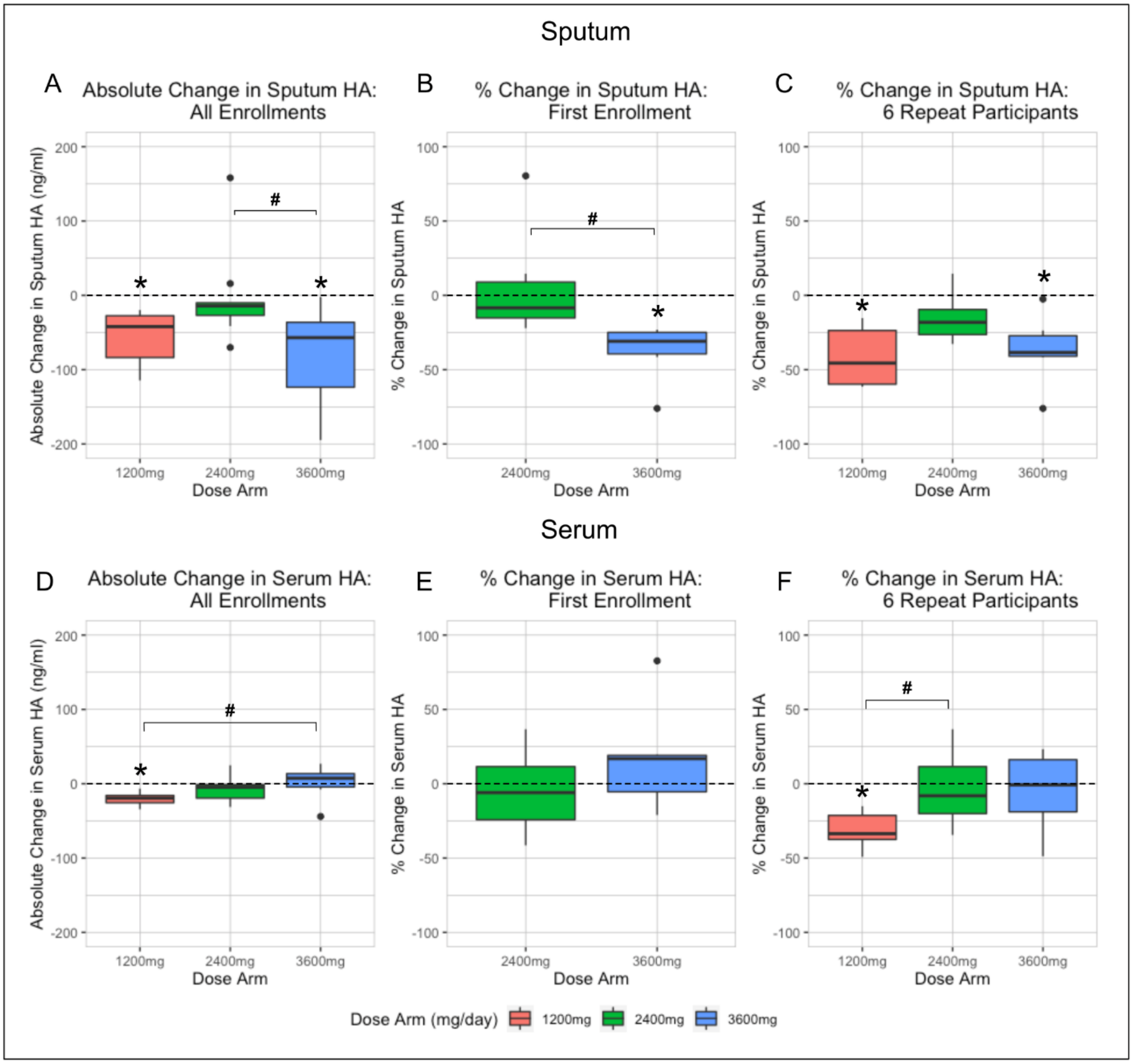

Supplemental Figure 1. Change in sputum and serum HA levels after 4-MU treatment. (A) Sputum absolute HA levels decreased significantly in the 1200 mg and 3600 mg arms. The decrease in the 3600 mg was significantly larger than in the 2400 mg arm. **(B)** In the analysis restricted to the first enrollment, the percent change of sputum HA in the 3600 mg was significantly larger than in the 2400 mg arm. **(C)** In the analysis restricted to the six repeat participants, sputum HA decreased significantly from baseline in the 1200 mg and 3600 mg arms; there was no significant difference between arms. **(D)** Serum absolute HA decreased significantly in the 1200 mg arm only; this was also significantly different from the 3600 mg arm. **(E)** In the analysis restricted to the first enrollment, serum HA did not change significantly from baseline in either arm. **(F)** In the analysis restricted to the six repeat participants, serum HA decreased significantly from baseline in the 1200 mg arm and this was significantly different from the 2400 mg arm. *Statistically significant difference from baseline to Day 4 of treatment by paired t-test. The dashed line indicates the baseline reference level. #Statistically significant difference between dose arms using unpaired t-test across all enrollments (**A** & **D**) and first enrollment (**B** & **E**) and paired t-tests across the 6 repeat participants (**C** & **F**). In panels **C** & **F** showing the comparison for participants who repeated all 6 doses, n = 6 in each arm. In all other panels (**A**, **B**, **D**, and **E**), the sample size is n = 8, n = 9, and n = 9 for the 1200 mg, 2400 mg, and 3600 mg arms respectively. Each boxplot represents the median, interquartile range, 1.5 times the interquartile range, and data points outlying the whisker range.

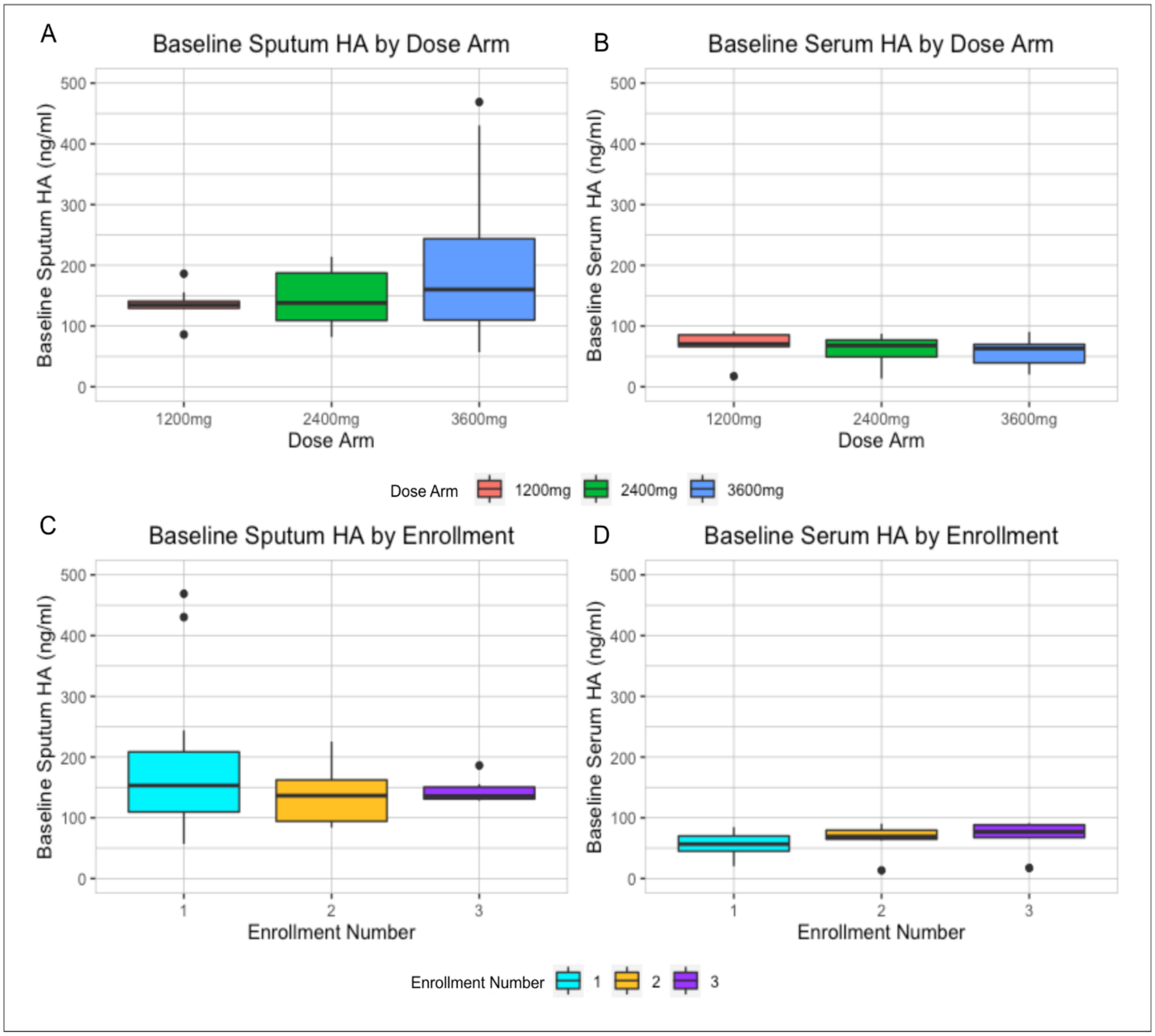

Supplemental Figure S2. Baseline sputum and serum HA by dose arm and enrollment number. (**A-B**) There were no statistically significant differences in baseline sputum or serum HA levels across dose arms by unpaired t-tests. (**C-D**) There were no statistically significant differences in baseline sputum or serum HA levels across different enrollments by unpaired t-tests. Each boxplot represents the median, interquartile range, 1.5 times the interquartile range, and data points outlying the whisker range.

Supplemental Table 1. Adverse Events

Adverse Event	# of Events / # of Participants n/N (%)	Unique Subjects with events (N)	Dose Group		
			1200 mg	2400 mg	3600 mg
Diarrhea	1/26 (3.8%)	1	0	0	1
Dizziness	1/26 (3.8%)	1	0	0	1
Elevated AST & ALT	1/26 (3.8%)	1	1	0	0
Headache	3/26 (11.5%)	2	0	1	2
Insomnia	2/26 (7.7%)	1	1	0	1
Nausea	1/26 (3.8%)	1	0	0	1

In total, nine adverse events were reported across all 26 enrollments. Two individuals experienced the same adverse event with two different doses (headache and insomnia).

Supplemental Table 2. Safety Labs

Variable	**Baseline** Mean +/- SD N = 26	**Day 4** Mean +/- SD N = 26	**Difference between Day 4 and Baseline** Mean difference (95% CI) N = 26
**CBC**			
WBC	5.93 ± 1.5	7.14 ± 1.66	1.24 (0.77 – 1.71)*
RBC	4.81 ± 0.38	4.87 ± 0.38	0.03 (-0.06 - 0.12)
Hemoglobin	14.08 ± 1.21	14.31 ± 1.25	0.17 (-0.1 - 0.44)
Hematocrit	42.98 ± 2.91	43.06 ± 3.01	-0.1 (-0.86 - 0.67)
Platelets	256.77 ± 56.93	257.04 ± 50.75	0.12 (-7.24 - 7.48)
Neutrophils ABS	3.44 ± 1.18	4.15 ± 1.31	0.71 (0.29 - 1.14)*
Lymphocytes ABS	1.87 ± 0.39	2.45 ± 0.63	0.58 (0.31 - 0.85)*
Monocytes ABS	0.43 ± 0.13	0.47 ± 0.12	0.04 (0.01 - 0.08)*
Eosinophils ABS	0.1 ± 0.09	0.13 ± 0.12	0.03 (0.01 - 0.06)*
Basophils ABS	0.05 ± 0.02	0.05 ± 0.02	0 (0 - 0.01)
**CMP**			
Sodium	139.92 ± 1.62	139.76 ± 1.76	-0.16 (-0.89 - 0.57)
Potassium	4.18 ± 0.33	3.95 ± 0.28	-0.24 (-0.43 - -0.06)*
Chloride	102.69 ± 1.54	102.76 ± 1.33	0.08 (-0.66 - 0.82)
CO2	25.81 ± 2.47	25.4 ± 2.02	-0.48 (-1.36 - 0.4)
BUN	14.04 ± 3.3	14.6 ± 3.55	0.56 (-0.47 - 1.59)
Creatinine	0.83 ± 0.18	0.79 ± 0.14	-0.05 (-0.1 - 0)
Glucose	83.38 ± 15.62	91.2 ± 15.98	8.36 (0.67 - 16.05)*
Calcium	9.51 ± 0.31	9.53 ± 0.29	0.01 (-0.1 - 0.13)
Protein total	7.32 ± 0.39	7.42 ± 0.46	0.08 (-0.09 - 0.26)
Albumin	4.6 ± 0.17	4.68 ± 0.26	0.09 (-0.01 - 0.19)
Bilirubin total	0.59 ± 0.41	0.58 ± 0.43	-0.02 (-0.11 - 0.06)
Alkaline Phosphatase	72.77 ± 16.88	73.6 ± 17.25	0.88 (-2.18 - 3.94)
AST	26.85 ± 7.23	56 ± 146.24	29.48 (-31.2 - 90.16)
ALT	24.81 ± 12.83	30.16 ± 31.38	5.44 (-7.98 - 18.86)
AST†	26.85 ± 7.23	26.79 + 7.81	0.13 (-3.42 – 3.67)
ALT†	24.81 ± 12.83	24.21 + 10.19	-0.92 (-3.84 – 2.01)

One individual had missing data for the CBC differential cell count. *Statistically significant difference between Day 4 follow-up and baseline on paired t-test at $p<0.05$. †Analysis excluding one enrollment with an adverse event of AST and ALT elevations.
